# Regulation of Lifespan, Metabolism, and Stress Responses by the *Drosophila* SH2B Protein, Lnk

**DOI:** 10.1371/journal.pgen.1000881

**Published:** 2010-03-19

**Authors:** Cathy Slack, Christian Werz, Daniela Wieser, Nazif Alic, Andrea Foley, Hugo Stocker, Dominic J. Withers, Janet M. Thornton, Ernst Hafen, Linda Partridge

**Affiliations:** 1 Institute of Healthy Ageing, Department of Genes, Evolution, and Environment, University College London, London, United Kingdom; 2 ETH Zurich, Institute of Molecular Systems Biology, Zurich, Switzerland; 3 European Bioinformatics Institute, Wellcome Trust Genome Campus, Hinxton, Cambridge, United Kingdom; 4 Centre for Diabetes and Endocrinology, Rayne Institute, University College London, London, United Kingdom; Stanford University School of Medicine, United States of America

## Abstract

*Drosophila* Lnk is the single ancestral orthologue of a highly conserved family of structurally-related intracellular adaptor proteins, the SH2B proteins. As adaptors, they lack catalytic activity but contain several protein–protein interaction domains, thus playing a critical role in signal transduction from receptor tyrosine kinases to form protein networks. Physiological studies of SH2B function in mammals have produced conflicting data. However, a recent study in *Drosophila* has shown that Lnk is an important regulator of the insulin/insulin-like growth factor (IGF)-1 signaling (IIS) pathway during growth, functioning in parallel to the insulin receptor substrate, Chico. As this pathway also has an evolutionary conserved role in the determination of organism lifespan, we investigated whether *Lnk* is required for normal lifespan in *Drosophila*. Phenotypic analysis of mutants for *Lnk* revealed that loss of *Lnk* function results in increased lifespan and improved survival under conditions of oxidative stress and starvation. Starvation resistance was found to be associated with increased metabolic stores of carbohydrates and lipids indicative of impaired metabolism. Biochemical and genetic data suggest that Lnk functions in both the IIS and Ras/Mitogen activated protein Kinase (MapK) signaling pathways. Microarray studies support this model, showing transcriptional feedback onto genes in both pathways as well as indicating global changes in both lipid and carbohydrate metabolism. Finally, our data also suggest that *Lnk* itself may be a direct target of the IIS responsive transcription factor, dFoxo, and that dFoxo may repress *Lnk* expression. We therefore describe novel functions for a member of the SH2B protein family and provide the first evidence for potential mechanisms of SH2B regulation. Our findings suggest that IIS signaling in *Drosophila* may require the activity of a second intracellular adaptor, thereby yielding fundamental new insights into the functioning and role of the IIS pathway in ageing and metabolism.

## Author Summary

Many human populations are experiencing increased life expectancy, and as populations age the incidence of age-related diseases becomes more prevalent. The identification of single gene mutations that extend lifespan in invertebrate model organisms has revealed that several cellular signaling pathways, including the insulin/insulin-like growth factor (IGF)-1 signaling (IIS) pathway, play a crucial role in modulating the ageing process across multiple species. Thus, studies carried out in yeast, worms, and flies have revealed evolutionarily conserved mechanisms of ageing, which are likely to be relevant to mammals, including humans. A recent study in *Drosophila* identified the SH2B family adaptor protein, Lnk, as an important regulator of the IIS pathway during organismal growth. In this study, we show that Lnk is also required to determine normal lifespan in *Drosophila*, as mutations that disrupt *Lnk* activity result in increased lifespan. In addition, these mutants show improved survival under conditions of stress and metabolic disregulation. Furthermore, we show that the expression of *Lnk* is regulated by the IIS responsive transcription factor, dFoxo. Our data therefore provide new mechanistic insights into the role of the IIS pathway in ageing.

## Introduction

SH2B proteins are a recently identified family of intracellular adaptor proteins that transduce signals downstream of a number of receptor tyrosine kinases (RTKs). These include the receptors for insulin, insulin-like growth factor-1, Janus kinase 2 (Jak2), platelet derived growth factor, fibroblast growth factor and nerve growth factor [1]–[5]. Consequently, SH2B proteins have been shown to function during multiple physiological processes including glucose homeostasis, energy metabolism, hematopoesis and reproduction [6]–[9]. Moreover, mutations in SH2B orthologues in humans are associated with metabolic disregulation and obesity. Several SH2B family members have been identified in mammals so far including SH2B1 (of which there are four splice variants: SH2B1α, SH2B1β, SH2B1γ and SH2B1δ), SH2B2 (APS) and SH2B3 (Lnk). They are characterised by a number of conserved domains including a central pleckstrin homology (PH-) domain, a C-terminal Src Homology 2 (SH2-) domain, an N-terminal proline rich region, multiple consensus sites for tyrosine and serine/threonine phosphorylation and a highly conserved C-terminal c-Cbl recognition motif [6], [10]–[12]. These domains function as protein-protein interaction motifs and so allow SH2B proteins to integrate and transduce intracellular signals from multiple signaling networks in the absence of intrinsic catalytic activity [6], [10]–[12].

Biochemical studies have demonstrated that SH2B proteins bind via their SH2 domains to phosphotyrosine residues within the intracellular tails of several activated RTKs thereby contributing to receptor activation [10],[13],[14]. Once bound, SH2B proteins have been shown to undergo RTK-stimulated tyrosine phosphorylation although they might also be serine/threonine phosphorylated in their basal state as they show anomalous migration on SDS/PAGE indicative of protein structural modifications [13],[15],[16]. *In vitro* binding assays have identified interactions between SH2B proteins and a number of other intracellular adaptor proteins including the insulin receptor substrates IRS1 and IRS2, Grb2, Shc and c-Cbl [2],[17],[18]. These interactions may or may not require tyrosine phosphorylation of SH2B depending on the isoform studied [2],[18]. Interactions with IRS proteins promote activation of the phosphoinositol-3 kinase (PI3K) pathway and overexpression in cell culture has been show to enhance activation of both the PI3K and the Ras/MapK pathways [17],[19]. Binding to the proto-oncogene product, c-Cbl, a RING-type E2-dependent ubiquitin protein ligase, may facilitate either endocytosis or degradation of the receptor through receptor ubiquitination [16],[20]. Thus, SH2B proteins may have dual functionality in both positively and negatively regulating RTK signaling.

Mammalian SH2B family members are widely expressed in a number of tissues suggesting that they may share some overlapping, redundant functions [13],[21],[22]. For example, mice carrying a genetic deletion for SH2B3 show a selective defect in the regulation of B cell lymphopoeisis. This is consistent with the high levels of SH2B3 expression observed in hematopoetic organs such as the bone marrow and lymph nodes [22] and suggests that SH2B3 plays a specific, non-redundant role in the development of a subset of immune cells. However, SH2B3 mRNA is also abundant in non-hematopoetic tissues such as testis, brain and muscle and so presumably the absence of phenotype in these tissues indicates redundancy with other SH2B family members [22]–[24]. Studies into the physiological functions of SH2B1 and SH2B2 have produced contradictory results. Genetic deletion of SH2B1 in mice produces neonatal growth retardation and infertility probably due to impaired responses to GH or IGF-1 [7]. It was reported that SH2B1 null mice rapidly increase their body mass and develop obesity as a result of significantly impaired hypothalamic leptin signaling resulting in hyperleptinemia and hyperphagia [8],[25]. These mice were also shown to have attenuated insulin signaling in muscle, liver and fat resulting in insulin resistance and diabetes. More recently, a second model showed that SH2B1 null mice actually have decreased fat mass possibly caused by a reduction in adipogenesis as SH2B1 deficiency was associated with reduced expression of adipogenic genes such as peroxisome proliferator-activated receptor γ (PPARγ) and impaired adipocyte differentiation in cell culture [26]. In the case of SH2B2, it was reported that SH2B2 null mice develop hypoinsulinemia and show increased insulin sensitivity at young ages [27]. However, more recent reports saw no effect of SH2B2 deletion on fasted blood glucose, insulin levels, glucose or insulin tolerance [9]. The reasons for this apparent discrepancy between studies is unclear but may be confounded by differences in genetic backgrounds, diet or housing conditions.

Understanding the physiological functions of SH2B proteins in mammals has therefore been complicated by the presence of multiple SH2B isoforms and conflicting data from genetic analyses. The genome of *Drosophila melanogaster* encodes a single SH2B homologue (*Lnk*) that shares a similar domain structure to its mammalian counterparts, with 36% sequence identity to human SH2B proteins in its PH-domain and 74% sequence identity in its PTB domain as well as containing a highly conserved c-Cbl binding motif. Furthermore, most of the basic metabolic and signaling pathways that maintain homeostasis are conserved in the fly providing an ideal context for *in vivo* studies of SH2B biological function.

Recent evidence has shown that *Drosophila* Lnk is a key regulator of cell growth and proliferation during development [28]. Loss-of-function mutations in *Lnk* produce phenotypes reminiscent of reduced IIS signalling such as growth reduction, developmental delay and female sterility. Genetic epistasis experiments indicated that Lnk functions downstream of the *Drosophila* Insulin Receptor (dInR*)* and upstream of PI3K in IIS-mediated growth control. Genetic epistasis suggested that Lnk may play a similar role as the insulin receptor substrate, Chico, in the activation of PI3K upon dInR stimulation during growth [28]. Mutations that reduce IIS activity in *C. elegans*, *Drosophila* and mouse can increase lifespan in all three organisms, demonstrating that the IIS pathway has evolutionary conserved roles in the determination of adult lifespan. In *Drosophila*, the effects of insulin receptor activity on lifespan determination are mediated via the Chico/PI3K/forkhead transcription factor [29]–[32]. Therefore, we investigated whether Lnk also plays a role in the determination of adult lifespan. Here, we show that *Lnk* mutant flies exhibit increased lifespan as well as improved survival under conditions of oxidative stress and starvation. We also show that *Lnk* loss-of-function results in increased stored energy reserves associated with transcriptional changes in genes involved in both lipid and carbohydrate metabolism. Biochemical and genetic data indicate that Lnk functions within both the IIS and Ras/MapK signaling cascades and is itself a direct target for transcriptional regulation by the dFoxo transcription factor.

## Results

### *Lnk* mutants have increased lifespan

**Figure 1:**
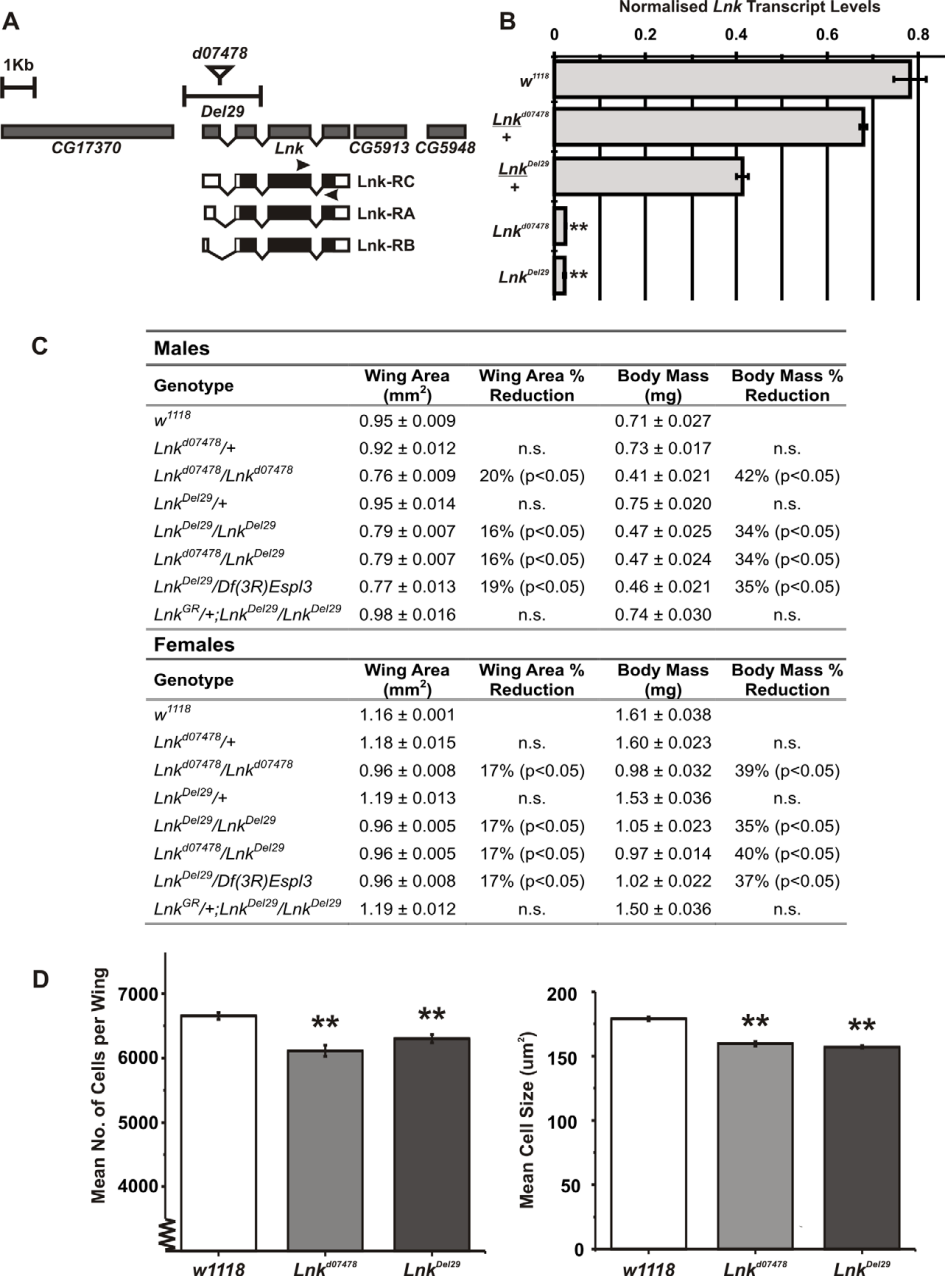
*Lnk* loss-of-function mutations reduce body size. (A) Schematic representation of the *Lnk* locus along with the flanking genes *CG17370, CG5913* and *CG5948*. The three *Lnk* transcripts (RA, RB, and RC) are shown along with the positions of the P-element insertion *d07478* and the deletion *Del29*. Arrows show the position of primers used for the quantitative RT–PCR in (B). (B) Quantitative RT–PCR analysis of *Lnk* transcript expression in 7-day old female flies. *Lnk* transcripts were amplified using the primers indicated in (A) and normalised to *actin5C.* (C) Mean wing area and body mass for male and female flies of the indicated genotypes. Data are represented as means ± SEM (n = 10 for each measurement). Percentage differences compared to *w^1118^* controls are indicated (n.s.  =  not significantly different). (D) The wings of homozygous *Lnk* mutant females contain fewer and smaller cells compared to the *w^1118^* controls. Data are shown as means ±SEM (n = 10). ** denotes statistically significant difference (*p*<0.05).

Novel alleles of *Lnk* were recently isolated in a genetic screen looking for new regulators of growth in flies as *Lnk l*oss-of-function clones were found to cause cell-autonomous growth inhibition in the developing eye [28]. We have characterised two additional mutant alleles of *Lnk*: *Lnk^d07478^* containing a P-element insertion within the first intron of the *Lnk* locus and *Lnk^Del29^*, a small deletion generated by FLP-FRT recombination between two pBAC elements that removes the first two exons of *Lnk* including the predicted translational start site (Figure 1A). Homozygous mutants had significantly reduced levels of *Lnk* transcripts as measured by quantitative RT-PCR (Figure 1B). Homozygous and transheterozygous mutants under normal culture conditions were adult viable but developmentally delayed with an overall reduction in body size as a result of reduced cell size and cell number (Figure 1C and 1D). No further reductions in growth were observed in hemizygous combinations over a deficiency that removes the entire *Lnk* locus suggesting that they represent strong loss-of-function alleles (Figure 1C). In addition, we were able to fully rescue the growth defects of *Lnk^Del29^* homozygotes by introducing a genomic rescue construct containing the entire *Lnk* locus indicating that these growth defects are specific to *Lnk* (Figure 1C).

**Figure 2:**
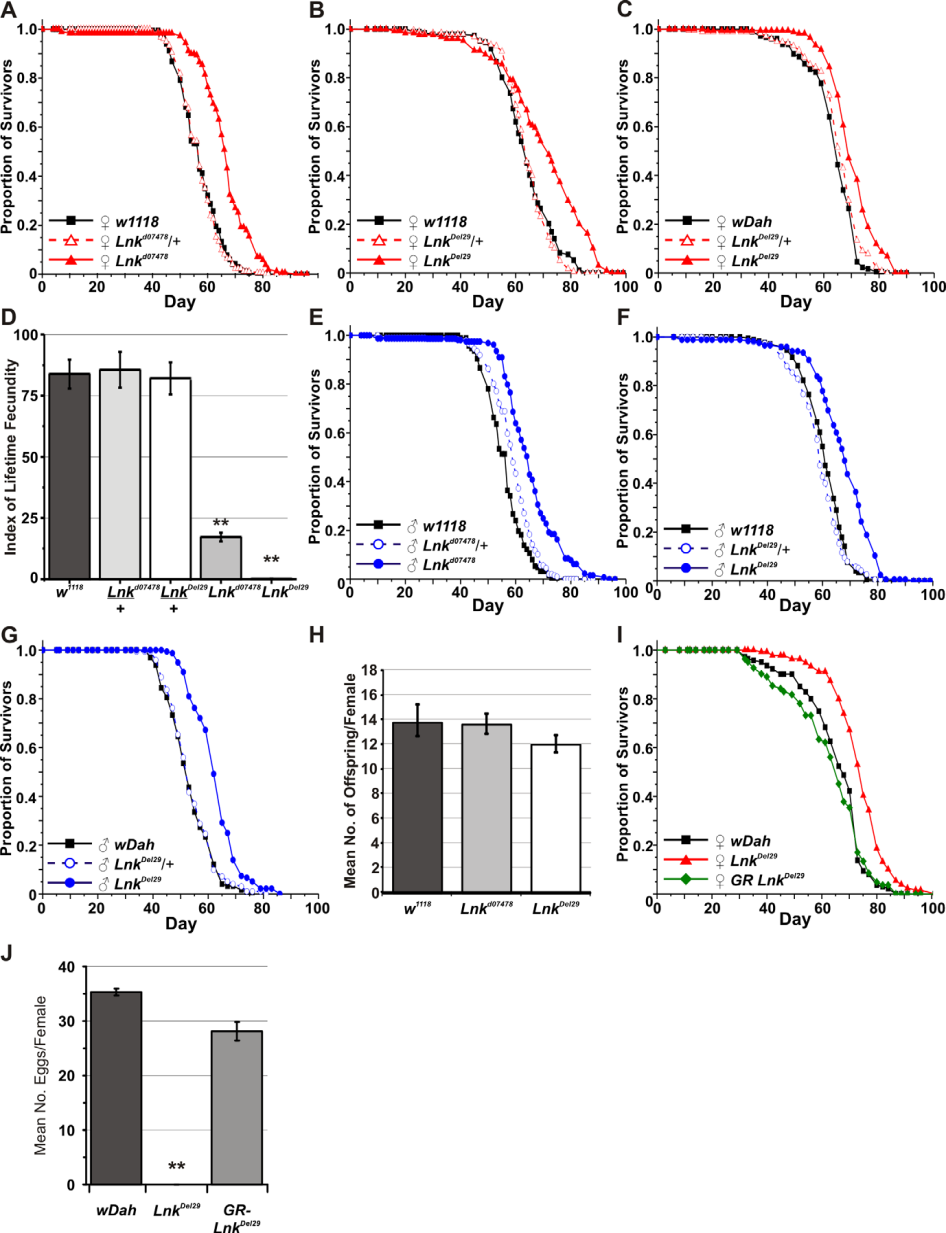
Mutation of *Lnk* extends lifespan in both sexes and reduces female fecundity. (A) *w^1118^*-backcrossed females. Median lifespans are: 57 days for *w^1118^* (n = 196), 58 days for *Lnk^d07478^/+* (n = 203) and 65 days for *Lnk^d07478^/Lnk^d07478^* (n = 163). Log rank test χ^2^ and *p-*values: *w^1118^* versus *Lnk^d07478^/+* (χ^2^ = 1.7, *p = *0.192) and *w^1118^* versus *Lnk^d07478^/Lnk^d07478^* (χ^2^ = 90.2 , *p<*0.0001). (B) *w^1118^*-backcrossed females. Median lifespans are 61 days for *w^1118^* (n = 173), 59 days for *Lnk^Del29^/+* (n = 200) and 68 days for *Lnk^Del29^/Lnk^Del29^* (n = 182). Log rank test χ^2^ and *p-*values: *w^1118^* versus *Lnk^Del29^/+* (χ^2^ = 2.2, *p = *0.139) and *w^1118^* versus *Lnk^Del29^/Lnk^Del29^* (χ^2^ = 65.2, *p<*0.0001). (C) *w^Dah^*-backcrossed females. Median lifespans are 63 days for *w^Dah^* (n = 179), 66 days for *Lnk^Del29^/+* (n = 177) and 68 days for *Lnk^Del29^/Lnk^Del29^* (n = 178). Log rank test χ^2^ and *p-*values: *w^Dah^* versus *Lnk^Del29^/+* (χ^2^ = 4.67, *p = *0.03) and *w^Dah^* versus *Lnk^Del29^/Lnk^Del29^* (χ^2^ = 53.09, *p<*0.0001). (D) Reduced fecundity in *Lnk* homozygous mutant females. Index of lifetime fecundity represents the mean number of eggs laid per female per day at 10 time points during the first 40 days of life. Index of lifetime fecundity in *Lnk* homozygous mutant females (white bars) is significantly reduced compared to control flies (*w1118*; dark grey bars) and heterozygotes (light grey bars). Data are shown as means ±SEM. ** denotes statistically significant difference (*p*<0.05). (E) *w^1118^*-backcrossed males. Median lifespans are 57 days for *w^1118^* (n = 198), 57 days for *Lnk^d07478^/+* (n = 196) and 67 days for *Lnk^d07478^/Lnk^d07478^* (n = 190). Log rank test χ^2^ and *p-*values: *w^1118^* versus *Lnk^d07478^/+* (χ^2^ = 0.1 , *p = *0.778) and *w^1118^* versus *Lnk^d07478^/Lnk^d07478^* (χ^2^ = 107.0, *p<*0.0001). (F) *w^1118^*-backcrossed males. Median lifespans are 63 days for *w^1118^* (n = 192), 63 days for *Lnk^Del29^/+* (n = 181) and 71 days for *Lnk^Del29^/Lnk^Del29^* (n = 144). Log rank test χ^2^ and *p-*values: *w^1118^* versus *Lnk^Del29^/+* (χ^2^ = 0.5, *p = *0.463) and *w^1118^* versus *Lnk^Del29^/Lnk^Del29^* (χ^2^ = 37.0, *p<*0.0001). (G) *w^Dah^*-backcrossed males. Median lifespans are 52 days for *w^Dah^* (n = 169), 52 days for *Lnk^Del29^/+* (n = 168) and 61 days for *Lnk^Del29^/Lnk^Del29^* (n = 171). Log rank test χ^2^ and *p-*values: *w^Dah^* versus *Lnk^Del29^/+* (χ^2^ = 0.24, *p = *0.627) and *w^Dah^* versus *Lnk^Del29^/Lnk^Del29^* (χ^2^ = 44.89, *p<*0.0001). (H) Mean number of eggs laid per *w^1118^* female crossed to males of the indicated genotype. Data are shown as mean number of eggs laid per female fly over a four day period ± SEM. (I) Genomic rescue (GR) of *Lnk* in *w^Dah^*-backcrossed females. Median lifespans are 67 days for *w^Dah^* (n = 151), 74 days for *Lnk^Del29^/Lnk^Del29^* (n = 150) and 66 days for *LnkGR;Lnk^Del29^/Lnk^Del29^* (n = 92). Log rank test χ^2^ and *p-*values: *w^Dah^* versus *Lnk^Del29^/Lnk^Del29^* (χ^2^ = 33.87, *p<*0.0001) and *w^Dah^* versus. *LnkGR;Lnk^Del29^/Lnk^Del29^* (χ^2^ = 0.71, *p = *0.40). (J) Genomic rescue of female fertility defects. Eggs were counted on day 7 of the lifespan experiment shown in (I). Data are shown as mean number of eggs/female over a 24 hour period ± SEM. ** denotes statistically significant difference (*p*<0.05).

Both alleles were backcrossed for more than eight generations into two distinct genetic backgrounds: the inbred *w^1118^* strain and the outbred *w^Dahomey^ (w^Dah^)* strain. We then assayed heterozygotes and homozygotes of both alleles for longevity. After backcrossing into the *w^1118^* genetic background, heterozygosity for either *Lnk^d07478^* or *Lnk^Del29^* did not result in any significant differences in lifespan in either males or females (Figure 2A, 2B, 2E, and 2F). In contrast, we observed significant increases in both median and maximum lifespan in both males and females homozygous mutant for either allele when compared to wild-type controls (Figure 2A, 2B, 2E, and 2F). Furthermore, the longevity effects of *Lnk^Del29^* males and females were fully reproducible after backcrossing into *w^Dah^* (Figure 2C and 2G) and the lifespan extension observed in females homozygous mutant for *Lnk^Del29^* was fully rescued by the introduction of a *Lnk* genomic rescue construct (Figure 2I), thereby confirming a role for *Lnk* in lifespan determination.

In addition to increased lifespan, homozygous *Lnk* females produced significantly fewer eggs compared to their wild-type counterparts, especially *Lnk^Del29^* homozygous females, which were practically sterile (Figure 2D). Furthermore, *Lnk* mutant ovaries were dramatically reduced in size and contained immature oocytes that were arrested in previtellogenic stages of oogenesis (data not shown) and the egg laying defects observed in *Lnk^Del29^* homozygous females were fully rescued in the presence of a *Lnk* genomic rescue construct (Figure 2J). We observed no obvious defects in the fertility of *Lnk* homozygous males and females mated to *Lnk* mutant males produced comparable numbers of eggs as females mated to *w^1118^* males (Figure 2H).

### *Lnk* mutants are stress resistant and show metabolic disregulation

**Figure 3:**
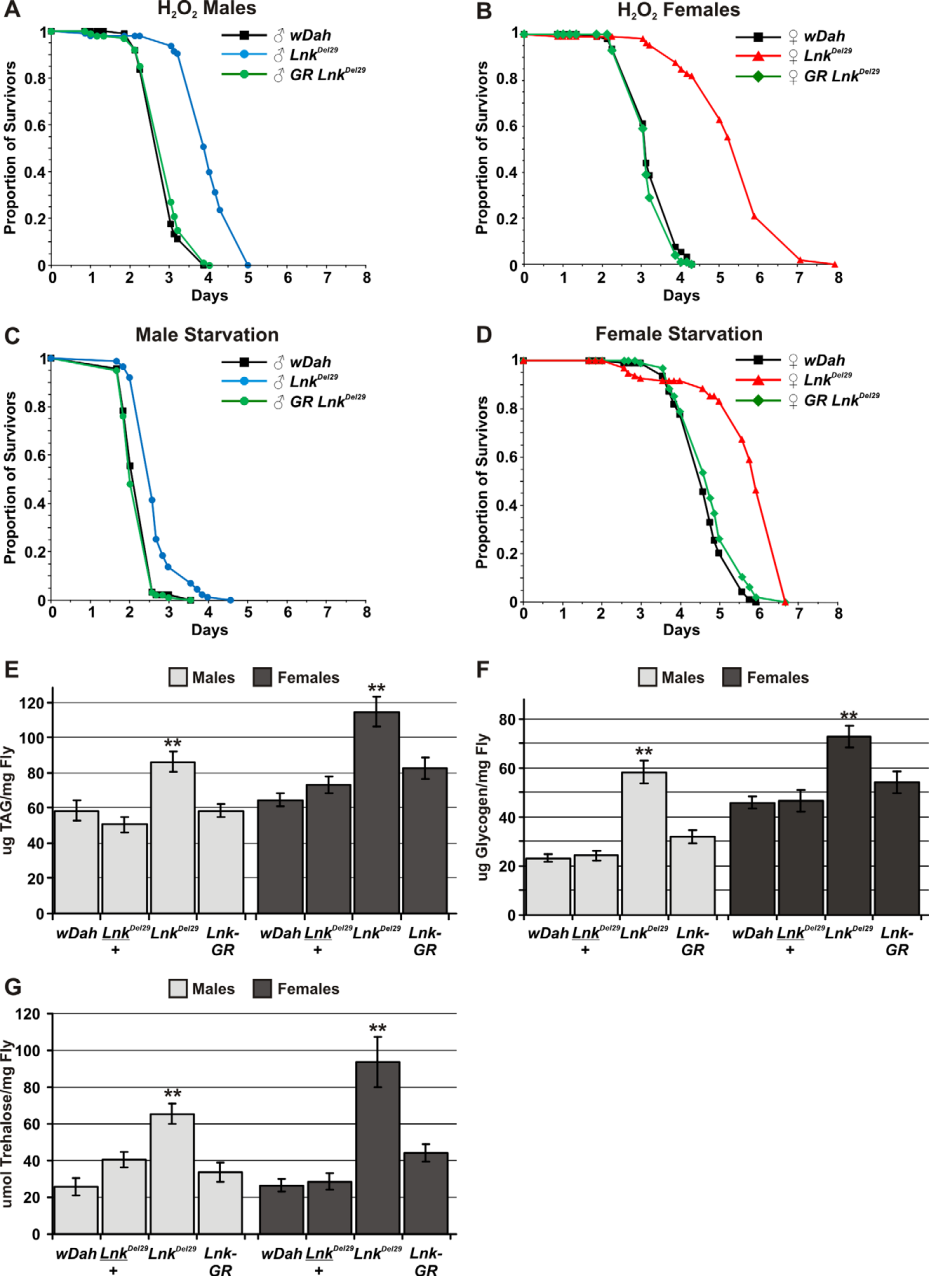
*Lnk* mediates responses to oxidative stress and starvation and is required for metabolic regulation. (A) Survival of male flies fed 5% hydrogen peroxide. *Lnk* mutant males live approximately 50% longer than controls and mutants carrying a *Lnk* genomic rescue (GR) construct (*p*<0.001). Median survival times are 2.6 days for *w^Dah^* (black line; n = 97), 3.9 days for *Lnk^Del29^* (blue line; n = 93) and 2.6 days for *LnkGR; Lnk^Del29^/Lnk^Del29^* (green line; n = 100). (B) Survival of female flies fed 5% hydrogen peroxide. *Lnk* mutant females live approximately 50% longer than controls and mutants carrying a *Lnk* genomic rescue (GR) construct (*p*<0.001). Median survival times are 3.1 days for *w^Dah^* (black line; n = 93), 5.6 days for *Lnk^Del29^* (red line; n = 105) and 3.1 days for *LnkGR; Lnk^Del29^/Lnk^Del29^* (green line; n = 100). (C) Survival of male flies under starvation conditions. *Lnk* mutant males live 30% longer than controls and mutants carrying a *Lnk* genomic rescue (GR) construct (*p*<0.001). Median survival times are as follows: 2.0 days for *w^Dah^* (black line; n = 92), 2.6 days for *Lnk^Del29^* (blue line; n = 86) and 1.9 days for *LnkGR; Lnk^Del29^/Lnk^Del29^*(green line; n = 97). (D) Survival of female flies under starvation conditions. *Lnk* mutant females live 35% longer than controls and mutants carrying a *Lnk* genomic rescue (GR) construct (*p*<0.001). Median survival times are as follows: 4.3 days for *w^Dah^* (black line; n = 94), 5.8 days for *Lnk^Del29^* (red line; n = 95) and 4.7 days for *LnkGR; Lnk^Del29^/Lnk^Del29^* (green line; n = 95). (E) Whole-fly content of triglycerides (TAG) per mg of fly (fresh weight). Data are presented as means (n = 10) ± SEM. ** denotes statistically significant difference (*p*<0.05). (F) Whole-fly glycogen content per mg of fly (fresh weight). Data are presented as means (n = 10) ± SEM. ** denotes statistically significant difference (*p*<0.05). (G) Whole-fly trehalose content per mg of fly (fresh weight). Data are presented as means (n = 10) ± SEM. ** denotes statistically significant difference (*p*<0.05).

Interventions that extend lifespan are often associated with enhanced resistance to various stresses [29],[33]. We therefore tested the ability of *Lnk* mutant flies to survive under conditions of oxidative stress and starvation. To induce oxidative stress, flies were starved for 5 hours and then fed 5% hydrogen peroxide in a sucrose/agar media. Both males and females, homozygous mutant for *Lnk*, showed significantly increased median survival times when fed 5% hydrogen peroxide compared to control flies under an identical regime (Figure 3A and 3B). Furthermore, this increased resistance to hydrogen peroxide was fully rescued in both sexes upon introduction of the *Lnk* genomic rescue construct (Figure 3A and 3B). We also observed a significant increase in survival times when *Lnk* mutant males and females were maintained on an agar-only diet to induce starvation (Figure 3C and 3D). Again, the starvation resistance observed in *Lnk* mutants was fully rescued in both sexes in the presence of the *Lnk* genomic rescue construct (Figure 3C and 3D). Moreover, resistance to hydrogen peroxide and starvation were observed with both *Lnk* mutant alleles and in both genetic backgrounds (Figure S1).

Enhanced survival under conditions of starvation is often associated with increased levels of stored energy resources indicative of a disruption to metabolic homeostasis. In flies, metabolised nutrients are primarily stored as triglycerides (TAG) and glycogen in the fat body, the insect equivalent of the mammalian liver and white adipose tissue. We observed significantly elevated levels of both TAG and glycogen in whole-fly extracts of both males and females when we compared *Lnk* mutants to wild-type controls (Figure 3E and 3F). These elevated levels of TAG and glycogen were restored back down to those observed in wild-type flies in the presence of a *Lnk* genomic rescue construct (Figure 3E and 3F) Despite the observed differences in metabolic stores, we did not detect any obvious differences in the feeding behaviour of *Lnk* mutant flies compared to age-matched controls (Figure S2) suggesting that this increase in metabolic stores is unlikely to be mediated by increased feeding but by changes in cellular metabolism.

In addition to glycogen, adult insects possess a second metabolic pool of carbohydrate in the form of the disaccharide trehalose which is a major sugar in the fat body, thorax muscles and hemolymph and is rapidly consumed during certain energy-requiring activities such as flight. We found that whole-body levels of trehalose were also significantly increased in *Lnk* mutant males and females when compared to controls (Figure 3G) and again, these elevated levels of trehalose were restored to those observed in wild-type flies by the introduction of the *Lnk* genomic rescue construct (Figure 3G). However, when we measured trehalose levels in hemolymph extracted from either third instar or adult flies we found no significant differences between *Lnk* mutants and controls (data not shown). The total volume of hemolymph in an adult fly is extremely small (approximately 0.1 µl) and so the contribution of hemolymph trehalose to the total trehalose content can be regarded as negligible. Thus, the increase in trehalose content in whole fly extracts is almost certainly caused by increased tissue trehalose stores. Insect hemolymph also contains circulating glucose which is obtained from the diet and again, we found no significant differences in circulating glucose levels in *Lnk* mutants compared to controls (data not shown).

### Transcriptional changes in *Lnk* mutants

In order to investigate further the molecular mechanisms of Lnk function, we performed microarray studies comparing the transcriptome of homozygous *Lnk* mutants to controls. FlyAtlas, a microarray-based atlas of adult gene expression in multiple *Drosophila* tissues (http://www.flyatlas.org; [34]), shows that *Lnk* mRNA is widely expressed in the adult fly but that transcripts are particularly enriched in the central nervous system. We therefore performed our transcriptome analysis on RNAs extracted from the heads of control and *Lnk* homozygous mutant females. After extraction, RNAs were labelled and hybridised to Affymetrix *Drosophila* 2.0 microarrays. All experiments were conducted in quadruplicate to facilitate statistical analysis. The raw data files were background corrected and normalised using the R programming language (see Material and Methods).

Using biological annotation available through the Gene Ontology (GO), we analysed our dataset using Catmap analysis. Catmap assigns significance to functional categories based on their representation within a ranked list of differentially expressed genes. This generated a list of GO terms associated with genes that show altered expression in *Lnk* mutants compared to controls (Table S1). The majority of the downregulated GO terms are involved in the metabolism of carbohydrates, amino acids, lipids and fatty acids suggesting that cellular metabolic processes are downregulated in *Lnk* mutant animals. Among the most significant upregulated GO terms are many linked to signal transduction and transcription indicating that these processes are upregulated in *Lnk* mutants compared to controls.

We then determined gene expression changes in our data set using a linear model. This study revealed that 2483 transcripts show significant differential expression (p<0.05; >0.1-fold) between *Lnk* mutants and controls with 1768 genes showing increased expression and 715 genes with decreased expression (Table S2). We compared this data set to a previously reported list of 484 transcripts that function in *Drosophila* metabolic pathways [35] and found that a number of genes in our differentially expressed gene list overlap with genes that regulate carbohydrate and lipid catabolism (Table S3). Downregulated genes included genes encoding several enzymes of the glycolytic pathway and the mitochondrial β-oxidation pathway while genes involved in glycogen synthesis and lipid storage showed upregulated expression. These changes in gene expression are consistent with an overall metabolic switch from catabolism to synthesis/storage and are congruent with our findings that *Lnk* mutants show increased levels of metabolic stores.

**Figure 4:**
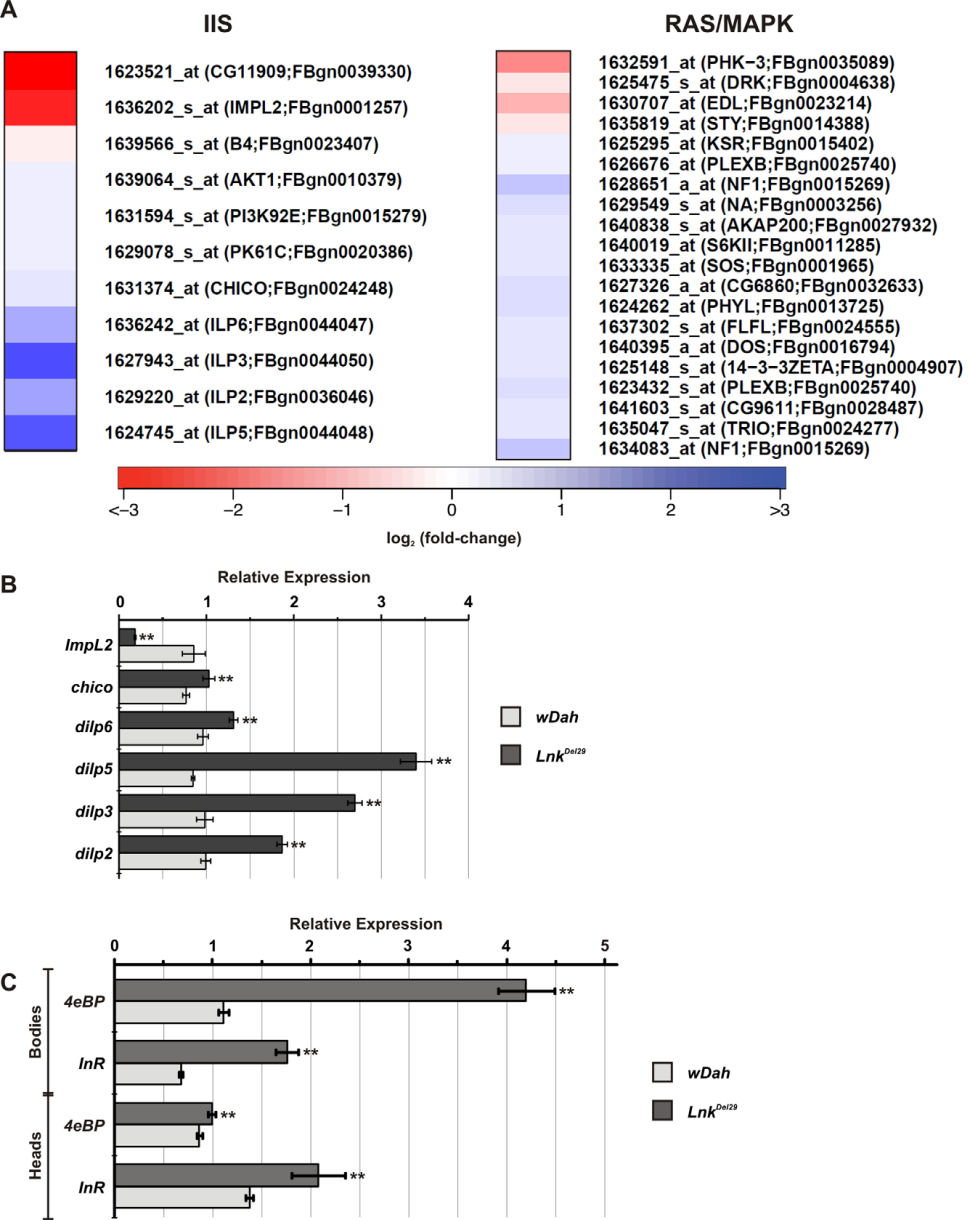
Regulation of signal transduction pathway gene expression in *Lnk* mutants. (A) Heat map showing expression of genes within the IIS and Ras/MapK signal transduction pathways that were significantly altered in *Lnk* mutants compared to controls. Red indicates lower expression; blue indicates higher expression (scale  =  log2 fold change). (B) Transcript levels of six IIS pathway genes measured by qRT-PCR and normalised to *actin5C*. RNA was extracted from adult female heads. Data are represented as mean normalised transcript level ± SEM (n = 4; ** *p*<0.05). (C) *4eBP* and *dInR* mRNA levels measured by qRT-PCR and normalised to *actin5C*. RNA was extracted from either heads or bodies of adult females. Data are represented as mean normalised transcript level ± SEM (n = 4; ** *p*<0.05).

Interestingly, a number of transcripts that function in the IIS pathway were found to be upregulated in *Lnk* mutants compared to controls. The mammalian SH2B proteins have been shown biochemically to function as intracellular adaptors for the mammalian insulin receptor and recent genetic data from *Drosophila* has shown that *Lnk* may play a similar role to *chico* during IIS-mediated growth control. We therefore compared our data set to a comprehensive list of transcripts that function in the IIS pathway in *Drosophila* (Figure 4A and Table S4). We found upregulation of transcripts encoding positive regulators of IIS including the insulin-like ligands *dilp2*, *dilp3*, *dilp5* and *dilp6* as well as *chico*, *Dp110*, *PDK-1* and *dAkt*. In contrast, we found downregulation of transcripts that encode negative regulators of the IIS pathway such as the IGFBP-like, *ImpL2,* and the PI3kinase inhibitor, *Susi*. Several of these IIS gene expression changes were further confirmed by quantitative RT-PCR (Figure 4B).

Transcriptional outputs of the IIS pathway are mediated via several downstream effectors including the forkhead transcription factor, dFoxo, the Ras/MapK signaling pathway and the protein kinase complex TORC1, all of which have been shown to regulate gene expression either directly or indirectly. Further examination of our microarray data set identified four known dFoxo target genes with upregulated expression: *split-ends (CG18497), ches-1-like (CG12690), eIF-4E (CG4035)* and *CG9009* (Table S4). In addition, we observed increased expression of two additional well-characterised dFoxo target genes, *4eBP* and *dInR* by qRT-PCR (Figure 4C). These data therefore suggest that dFoxo activity is increased in *Lnk* mutant flies.

Using EASE analysis followed by Fisher's exact test for statistical significance and Bonferroni correction for multiple comparisons, we found that IIS pathway genes and genes classified by Flybase in the functional category of Ras signal transduction were over-represented in our data set (*p = *0.002 and *p* = 0.004, respectively) (Table S4). In contrast, genes of the canonical TOR signaling pathway were not significantly over-represented in our data set (*p* = 0.784) (Table S4). Taken together, these data suggest significant transcriptional feedback onto the IIS via dFoxo and the Ras/MapK pathway but not via TOR signaling in *Lnk* mutant animals.

**Figure 5:**
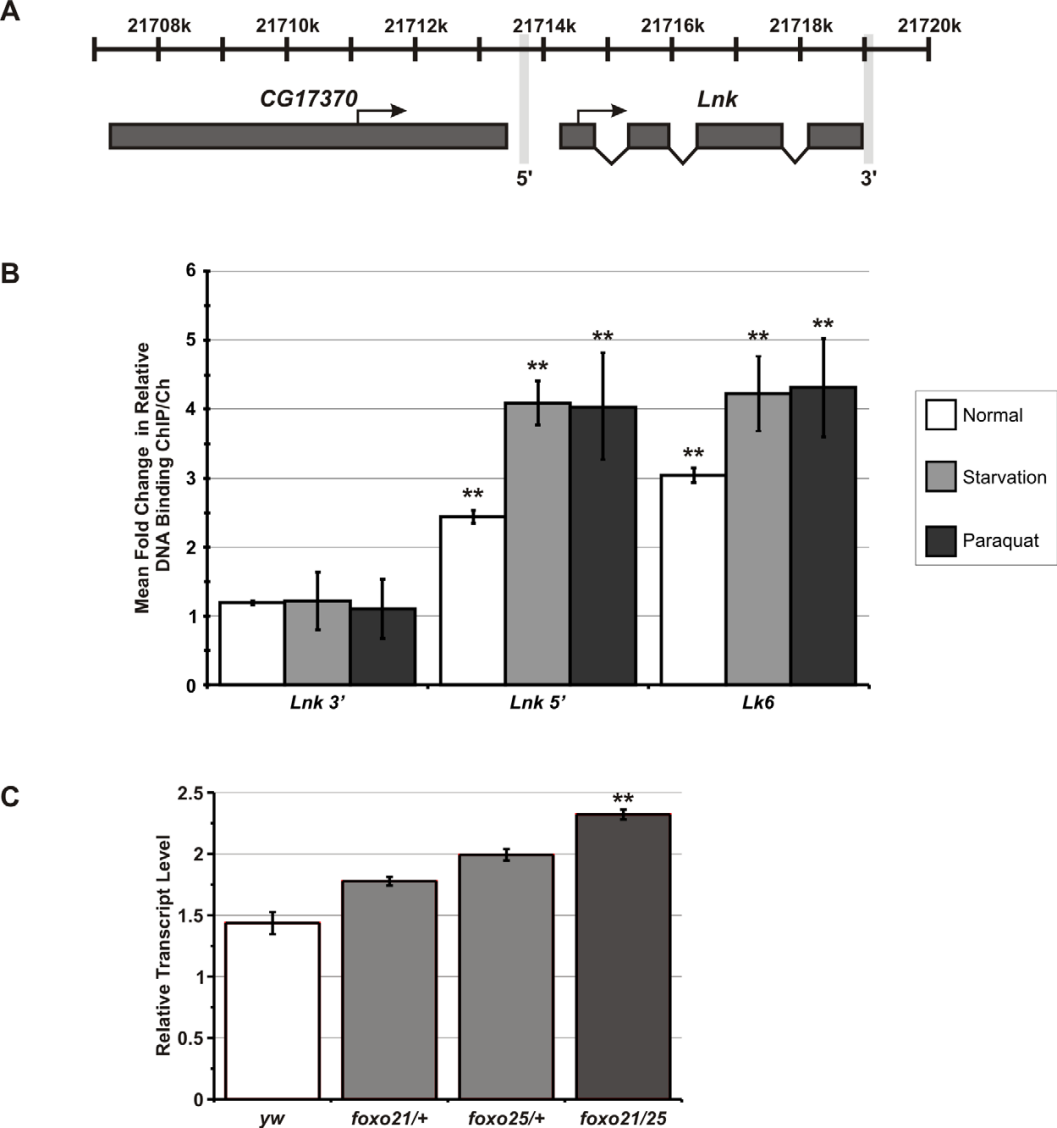
dFoxo binds to the promoter of *Lnk* and regulates *Lnk* expression. (A) Schematic diagram showing in vivo binding region for dFoxo at the *Lnk* locus as detected by chromatin immunoprecipitation (ChIP). Transcriptional start sites and the direction of transcription are indicated by arrows. The genomic region 3′ to the *Lnk* locus is also marked. (B) Quantitative PCR (qPCR) on the *Lnk* promoter, a 3′ region of the *Lnk* gene [as highlighted in (A)], the known dFoxo target gene, *Lk6* and the *U6* snRNA promoter (-87 to +61 from transcriptional start) to determine the proportion of DNA recovered after ChIP using anti-dFoxo antibody under normal, starvation and paraquat-treated conditions. Relative DNA binding was calculated as the proportion of chromatin recovered in the ChIP divided by that in the total chromatin preparation. Data are presented as mean fold-changes in relative DNA–binding compared to *U6* ± SEM of three biological repeats (** *p*<0.05). (C) *Lnk* mRNA levels in *dFoxo* mutant flies measured by qRT–PCR and normalised to *actin5C*. Data are represented as mean normalised transcript level ± SEM (n = 3; **p<0.05).

The potential for transcriptional feedback by dFoxo onto upstream components of the IIS cascade suggested that expression of *Lnk* itself may be regulated by dFoxo activity. We therefore looked for perfect matches to the mouse Foxo1/Foxo4 consensus binding site (RWWAACA) within 3Kb upstream of the *Lnk* translational start site and identified eight putative dFoxo binding sites in the *Lnk* promoter. To determine if dFoxo is indeed bound at the *Lnk* promoter, we performed chromatin immunoprecipitation (ChIP) using a specific dFoxo antibody [36]. Quantitative PCR (qPCR) was used to compare the relative DNA binding of dFoxo at a 5′ region of the *Lnk* promoter to two negative control genomic regions: a region within the *U6* snRNA promoter and a region 3′ to the *Lnk* locus just downstream of the last exon (Figure 5A). We observed a significant increase in the relative DNA binding at the *Lnk* promoter region compared to the negative controls (Figure 5B). The magnitude of this increase in relative DNA binding (approximately 2-fold) was comparable to that observed for *Lk6*, a known dFoxo target gene (Figure 5B). In addition, we observed further increases in dFoxo DNA-binding at both loci in flies that had been starved or treated with paraquat prior to chromatin extraction, conditions in which dFoxo is activated (Figure 5B). Furthermore, quantitative RT-PCR analysis of *Lnk* expression in RNA extracts from *foxo* mutant flies revealed that *Lnk* transcript levels are significantly elevated the absence of dFoxo (Figure 5C) suggesting that dFoxo may normally function to repress *Lnk* expression.

### Lnk as a component of the *Drosophila* IIS and Ras/MapK pathways

**Figure 6:**
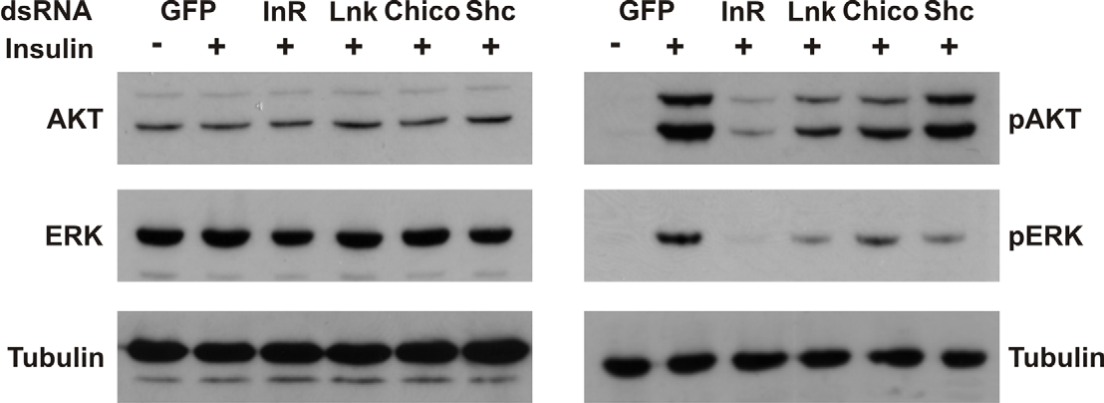
*Lnk* expression is required for insulin signaling and Ras/MapK signal transduction in flies. Western blot analysis of Akt and Erk-A phosphorylation in protein extracts of *Drosophila* S2 cells after treatment with the indicated dsRNAs before (-) and after (+) stimulation with human insulin. Knockdown of *dInR*, *chico*, or *Lnk* expression inhibits both Akt and Erk-A phosphorylation after insulin stimulation whereas knockdown of *Shc* inhibits phosphorylation of Erk-A only consistent with its role as an adapter for Ras/MAPK signaling. dsRNA against GFP was used as a negative control. Blots were probed with anti-tubulin as a loading control. Knockdowns of *Lnk* and *chico* transcripts were confirmed by Northern blot; knockdowns of *dInR* and *Shc* expression were confirmed by western blot (data not shown).

To assess the biological significance of a regulatory role for *Lnk* in the IIS and Ras/MapK pathways, we examined the effects of RNAi-mediated knockdown of *Lnk* expression on insulin-stimulated signaling in insect cells. Activation of the dInR by insulin triggers activation of both the PI3K and the Ras/MapK branches of the insulin signaling pathways resulting in the phosphorylation of various intracellular effectors, including Akt and the MapK, Erk-A [37]. RNAi-mediated knockdown of *Lnk* resulted in reduced levels of phosphorylated Akt and Erk-A upon insulin stimulation with no significant change in the levels of total protein (Figure 6). This reduction in phosphorylated Akt and Erk-A was comparable to that caused by RNAi-mediated knockdown of either the *dInR* or its intracellular substrate, *chico*, suggesting that *Lnk* expression is required for full insulin signaling transduction via both the PI3K and MAPK branches of the IIS pathway in cultured cells.

## Discussion

Our understanding of the physiological roles of the SH2B family of intracellular adaptors has been complicated by the presence of multiple family members in mammals. Furthermore, phenotypic analysis of genetic knockouts in mice has produced contradictory results. Recent genetic evidence has described a role for the single ancestral SH2B protein in *Drosophila* (Lnk) during IIS-mediated growth control. Here, we have characterised a critical role for Lnk in the regulation of lifespan, stress responses and cellular metabolism. Our results support a model in which Lnk functions as an intracellular adaptor for transduction of the IIS and Ras/MapK signaling cascades to mediate these physiological processes.

A recent genetic study has shown that mutations in *Drosophila Lnk* produce phenotypes reminiscent of reduced IIS during development including impaired growth, developmental delay and female sterility. Genetic epistasis experiments placed Lnk downstream of dInR and upstream of PI3K at the same level as Chico, the single fly insulin receptor substrate. Mutations in both *chico* and *Lnk* produce similar phenotypes and display similar reductions in IIS activity. Furthermore, flies homozygous mutant for both genes are lethal suggesting that they may be functionally redundant. The precise mechanisms whereby mammalian SH2B proteins transduce intracellular signaling from the insulin receptor remain unclear although like the IRS proteins, they have been shown to bind to multiple downstream mediators such as PI3K and Grb2 [6]. However, *Drosophila* Lnk lacks a consensus binding site for PI3K which is present in Chico so it is unlikely that they regulate similar downstream mechanisms.

The IIS pathway has an evolutionary conserved role in the determination of adult lifespan mediated by the Chico/PI3K/dFoxo branch of the IIS cascade. Previous studies have shown that flies either homozygous or heterozygous for *chico^1^*, a strong loss-of-function allele of *chico,* show increased lifespan [38]. We have shown that *Lnk* homozygotes also show increased lifespan although no obvious effects on lifespan were observed in heterozygous animals. Interestingly, the effects of *Lnk* mutation on lifespan extension were similar in both males and females, which is uncommon in *Drosophila,* even for IIS mutants. This data therefore suggests that as during growth regulation, signaling via the activated dInR during lifespan determination may require a second intracellular adaptor in addition to the insulin receptor substrate, Chico, and provides the first evidence of a role for SH2B proteins in lifespan determination.

Lifespan extension in females was associated with reduced fecundity as a result of an arrest in oogenesis. However, there were no visible effects of *Lnk* mutation on male fertility as measured by offspring production. As male homozygous mutants were also long-lived, this suggests that the extended lifespan of *Lnk* mutant females is not simply due to reduced fecundity. Genetic knockouts of SH2B1 in mice also show infertility due to impaired signal transduction from the IGF-1 receptor resulting in poor gonad development [7]. The sex-specific differences on fertility observed in *Lnk* mutants are probably due to sex-specific differences in *Lnk* transcript expression as microarray analyses of *Drosophila* gene expression has shown that *Lnk* transcripts are enriched within the female ovary but not in the male testis or accessory glands (http://www.flyatlas.org; [34]).

A comparison of the transcriptomes of *Lnk* mutant flies to controls revealed a number of gene expression changes associated with genes that encode components of the *Drosophila* IIS pathway. Hence, we observed upregulation of a number of factors that potentiate IIS such as the insulin-like ligands *dilp2*, *dilp3, dilp5 and dilp6*, as well as the insulin receptor substrate *chico*, the *Drosophila* class I PI3K, *Dp110*, phosphoinositide-dependent protein kinase *PDK-1* and *dAkt*. In contrast, the expression of negative regulators of IIS such as the IGFBP-like *ImpL2* and the PI3kinase inhibitor *susi* were downregulated. Several of these changes in expression were confirmed by qRT-PCR analysis and these data suggest that IIS transduction is affected by *Lnk* mutation, further strengthening the genetic evidence that *Lnk* is a component of the IIS pathway in flies. Transcriptional regulation downstream of IIS is in part mediated by the dFoxo transcription factor which is activated in response to low IIS by dAkt-mediated phosphorylation. While we did not observe any differences in dFoxo mRNA or protein levels in *Lnk* mutants compared to controls (data not shown), a number of dFoxo target genes did show changes in expression. Thus, *split-ends (CG18497), ches-1-like (CG12690), eIF-4E (CG4035)* and *CG9009* all showed upregulated expression in our microarray data set. We also observed increased expression of two well-characterised dFoxo target genes, *4eBP* and *dInR,* by quantitative RT-PCR. Taken together, these data suggest that dFoxo activity may be increased in *Lnk* mutant animals.

Interestingly, we observed a marked difference in the magnitude of increased expression of both *4eBP* and *dInR* between different body parts. Thus, for *4eBP* we observed a 1.1-fold increase in expression in head RNA extracts compared to a 3.8-fold increase in RNA extracts from bodies. Similarly, for *dInR* we observed a 1.5-fold increase in expression in head RNA extracts compared to a 2.6-fold increase in body RNA extracts. These data suggest that different tissues may exhibit differences in the magnitude of the transcriptional response to *Lnk* loss of function. As our microarray experiments were performed on RNA isolated from adult heads only, this may explain why *4eBP* and *dInR* were not identified in the microarray data set as microarray analysis of gene expression is generally regarded as less sensitive than qRT-PCR especially when changes in expression are small.

The observations that upstream components of the IIS pathway show transcriptional upregulation in response to *Lnk* loss of function suggest that transcriptional feedback back onto multiple components of the pathway may play an important regulatory role in IIS signal transduction. Previous studies have shown that *dInR* is itself a direct target of dFoxo so that when IIS levels are low, activated dFoxo increases *dInR* expression. In this study, we have shown that dFoxo also binds to the *Lnk* promoter *in vivo* suggesting that *Lnk* itself may be a direct target of dFoxo. dFoxo activity may also regulate transcription of IIS genes under basal conditions. Previous studies have shown that dFoxo is required for the basal expression of the *dilp3* ligand [39]. In our study, we found that in the absence of dFoxo, *Lnk* transcript expression increases suggesting that dFoxo activity is normally required for *Lnk* repression. Thus, regulation by dFoxo may involve both positive and negative effects on gene expression.

Our microarray data set also contained a number of differentially expressed genes that function within the Ras/MapK signal transduction pathway. Previous studies have shown that the Ras binding domain of *Drosophila* PI3K is required for maximal PI3K activity during growth and female egg laying linking Ras/MapK and IIS during growth and development in *Drosophila* [40]. Furthermore, we have shown that RNAi-mediated knockdown of *Lnk* inhibits insulin-stimulated Erk phosphorylation in insect cells. We cannot exclude the possibility that *Lnk* may play an adaptor function for Ras signaling downstream of other RTKs in addition to the insulin receptor. However, it should be noted that *Lnk* RNAi knockdown has no effect on Spitz-stimulated Erk phosphorylation via activation of the *Drosophila* EGF receptor [41].

Despite their small body size, *Lnk* mutants contain elevated levels of both lipid and carbohydrate stores. Consistent with their increased metabolic stores, *Lnk* mutants also showed increased survival under starvation conditions. Transcriptome analysis revealed gene expression changes in a number of components of metabolic regulation in *Lnk* mutants compared to controls. Thus, we observed reduced expression of several enzymes that function in the glycolytic pathway and upregulation of genes that function in glycogen synthesis. In addition, several genes in the mitochondrial β-oxidation pathway were downregulated whereas genes involved in the regulation of lipid storage showed increased expression. Taken together, these changes in gene expression are consistent with an overall inhibition of catabolic processes and upregulation of pathways that regulate the synthesis and storage of carbohydrates and lipids.

Studies on the metabolic defects of SH2B knockouts in mice have proved inconsistent. One group has shown that genetic deletion of SH2B1 impairs adipogenesis by downregulating adipogenic gene expression including PPARγ resulting in mice with decreased fat mass [26]. A *Drosophila* PPAR homolog has yet to identified but the closest *Drosophila* relative is the orphan receptor, E75 [42]. This gene was not among the differentially expressed gene list from our microarray data. Other studies have shown that SH2B1 null mice actually increase their body mass and develop obesity as a result of hyperphagia [8],[9]. In mammals, feeding is regulated by hypothalmic leptin signaling. Binding of leptin to its receptor results in receptor activation which in turn interacts with the non-receptor Janus kinase (Jak) stimulating downstream signaling events. Leptin stimulation of Jak is strongly potentiated by SH2B1 binding and so SH2B1 deletion impairs leptin signaling via Jak [4],[43],[44]. We did not observe any obvious differences in the feeding behaviour of *Lnk* mutant flies and there is no evidence to date that a leptin-like hormone exists in *Drosophila*. A functional Jak has been identified encoded by the *hopscotch* (*hop*) gene that has a well characterised role in hematopoesis in flies. We did not observe any obvious hematopoetic defects in *Lnk* mutants and *Lnk* was not found to genetically interact with any of the core JAK/STAT pathway components (data not shown). Our data therefore suggests that the increased adiposity in *Lnk* mutant flies is unlikely to be mediated by increased feeding or by defects in Jak signaling. In fact, our data suggest that the ancestral function of Lnk in *Drosophila* is to regulate carbohydrate and fat storage by regulating gene expression of several key metabolic regulatory pathways.

In mammalian cells, SH2B proteins have been shown to have dual functions during insulin signaling transduction by both activating and inhibiting downstream intracellular signaling events. Phosphorylation of SH2B2 by the activated insulin receptor creates a binding site for the proto-oncogene product c-Cbl. This promotes the ubiquitination of tyrosine kinase receptors by functioning as a RING-type E2-dependent ubiquitin protein ligase facilitating either endocytosis or proteasomal degradation of the receptor [16],[20]. The c-Cbl binding motif is conserved in *Drosophila* Lnk and so it will be of interest to determine whether the interaction with c-Cbl is important for Lnk function especially during lifespan regulation.

## Materials and Methods

### Fly stocks and husbandry

*w^1118^* and *Lnk^d07478^* were obtained from the Bloomington *Drosophila* Stock Centre. *yw*, *dfoxo*21a/TM3 and *dfoxo*25c*/*TM3 were a gift from the Hafen lab [45]. The *dfoxo*21a and *dfoxo*25c alleles were backcrossed for at least 6 generations into the *yw* background before use. The *Lnk^Del29^* deletion was generated using the pBAC insertions *Lnk^e01414^* and *Lnk^f02642^* (obtained from the Exelixis Collection at Harvard Medical School) according to published protocols [46]. A 6 kb fragment spanning from the 3′ end of *CG17370* to the beginning of the first exon of *CG5913* was used for the genomic rescue construct. This was inserted by means of ΦC31 mediated integration into a landing site on the second chromosome at 51D [28]. The wild-type stock Dahomey was collected in 1970 in Dahomey (now Benin) and has since been maintained in large population cages with overlapping generations on a 12L∶12D cycle at 25°C. The *white* Dahomey (*w^Dah^*) stock was derived by incorporation of the *w^1118^* deletion into the outbred Dahomey background by successive backcrossing. Both *w^1118^* and *w^Dah^* stocks were negative for the endosymbiont *Wolbachia* as determined by PCR using primers specific to *Wolbachia* genomic DNA. *Lnk* mutants were backcrossed for at least 8 generations into both *w^1118^* and *w^Dah^* genetic backgrounds before phenotypic analyses. Stocks were maintained and all experiments were conducted at 25°C on a 12h∶12h light:dark cycle at constant humidity using standard sugar/yeast/agar (SYA) medium [47]. For all experiments including lifespan experiments flies were reared at standard larval density and eclosing adults were collected over a 12 hour period. Flies were mated for 48 hours before sorting into single sexes.

### Body size measurements

Body weights of individual male and female 7-day old flies (n = 10 for each genotype) were measured using a precision balance. Wing areas, cell numbers and cell sizes were measured as previously described [48].

### Fertility tests

For female fecundity tests, female flies were housed with males for 48 hours post-eclosion and then separated into vials at a density of 5 or 10 females per vial. Eggs were collected over two 24-hour periods per week for 4 weeks. The number of eggs laid per vial at each time point was counted. For male fertility tests, individual 3 day old males were mated to 30 virgin females, 3 to 5 days of age. Matings were observed and then females were separated from the males and housed in vials at a density of 3 females per vial. Eggs were collected and counted over four consecutive 24 hour periods.

### Lifespan experiments

For lifespan experiments, flies were maintained in vials at a density of 10 flies per vial on standard SYA medium. Flies were transferred to new vials three times per week.

### Stress experiments

For all stress assays, flies were reared and housed as for lifespan experiments. For oxidative stress assays, 4-day old flies were first starved for 5 hours on 1% agar and then transferred onto 5% sucrose/agar containing 5% hydrogen peroxide. For starvation experiments, 7-day old flies were transferred to 1% agar.

### Feeding experiments

Feeding rates of flies were measured using a proboscis-extension assay in undisturbed conditions as previously described [49] using 7-day-old mated flies. Flies were housed at a density of 5 flies of the same sex per vial and transferred to new food on the evening before the assay. Feeding data is expressed as a proportion by experimental group (sum of scored feeding events divided by total number of feeding opportunities, where total number of feeding opportunities  =  number of flies in vial×number of vials in the group×number of observations). For statistical analyses, comparisons between experimental groups were made on the totals of feeding events by all flies within a vial, to avoid pseudoreplication.

### S2 cell culture and western blots

*Drosophila* S2 cell culture, dsRNA treatment and insulin treatment were as described in [37]. For western blots, 40 µg of total protein were resolved on 10% Tris-Glycine-SDS. Proteins were transferred to PVDF membranes and probed for total Akt (1∶1000; Cell Signaling), phospho-Akt (1∶1000; Cell Signaling), Erk (1∶1000; Cell Signaling), phospho-Erk (1∶1000; Cell Signaling) and tubulin (1∶5000; Sigma). Secondary antibodies conjugated to HRP were purchased from Biorad.

### Glucose and trehalose measurements

Hemolymph was collected and pooled from either 5 third instar larvae or 12 3-day old adult female flies. Glucose and trehalose levels were measured using the Glucose Infinity Reagent (ThermoScientific) as described in [33]. Whole fly trehalose in 7-day old adult males was measured as described in [33] and normalised to body weight.

### Glycogen and triglyceride measurements

Glycogen content of 7-day old adult males was measured as described in [33] and normalised to body weight. Levels of TAG in 7-day old adult males were measured using the Tryglyceride Infinity Reagent (ThermoScientific) and also normalised to body weight.

### Transcript expression analysis

Total RNA was extracted from 10 whole adult flies, 10 adult bodies or 25 adult heads per genotype using standard Trizol (Invitrogen) protocols. cDNA was prepared using oligod(T) primer and Superscript II reverse transcriptase according to the manufacturer's protocol (Invitrogen). Quantitative RT-PCR was performed using the PRISM 7000 sequence-detection system and Power SYBR® Green PCR Master Mix (ABI). Relative quantities of transcripts were determined using the relative standard curve method and normalized to *actin5C*. Three or four independent RNA extractions were used for each genotype. Primer sequences are available upon request.

### Microarray data analyses

Whole organism microarray experiments are generally only useful for detecting concerted changes of expression of widely expressed genes and most tissues will be under-represented in the array signal from a whole fly. Further complications arise from whole organism arrays when there are significant structural differences between treatments. *Lnk* transcripts are widely expressed but are particularly enriched within the central nervous system and as *Lnk* mutant ovaries show significant structural differences compared to controls, we restricted our microarray expression analysis to isolated heads.

Raw data (cel files) were processed to correct for probe-sequence biases, and R's implementation of the Affymetrix's MicroArray Suite 5.0 software was used to determine present target transcripts [50]. A transcript was considered present if the p-value was <0.111, and absent otherwise. The data was normalized using loess normalization and a linear model was fitted to identify a set of differentially expressed genes using the R limma package [51]. All individual probes have been mapped against all known and predicted transcripts of the *Drosophila melanogaster* genome release version 5.4. Promiscuous (some or all probes within a probe set map to more than one gene in the genome) and orphan (no probes in the probe set map to any known or predicted gene in the genome) probe sets were excluded from further analysis. FlyBase gene ids were mapped to Gene Ontology (GO) ids (version 1.107).

For functional analysis using all expressed genes, we used the Wilcoxon rank sum test implemented in Catmap [52]. Ranks of genes were based on the Bayes t-statistic for differential expression and, for a given functional category, the significance of the rank sum for all genes in the category was calculated analytically based on a random gene-rank distribution.

### Identification of dFoxo binding sites in the *Lnk* promoter

Sequence analysis was performed using Regulatory Sequence Analysis Tools [53] looking for perfect matches to the mouse Foxo1/Foxo4 binding sites [RWWAACA] [54].

### Chromatin Immunoprecipitation (ChIP)

Chromatin immunoprecipitations were carried out essentially as described by [55]. For starvation and paraquat treatments, flies were either starved for 24 hours or fed 20 mM paraquat for 16 hours. 1000 adult female flies were crushed to a fine powder under liquid nitrogen and suspended in 6 ml of PBS supplemented with Protease Inhibitor Cocktail (Sigma). Cross-linking was performed with 0.5% formaldehyde for 10 minutes and quenched by the addition of 1.5 ml of 2.5M glycine. The cross-linked chromatin was recovered by centrifugation and washed twice with FA/SDS buffer (50 mM Hepes-KOH, 150 mM NaCl, 1 mM EDTA, 0.1% Na Deoxycholate, 0.1% SDS, 1% Triton-X100 and 1 mM PMSF). Samples were resuspended in FA/SDS buffer and incubated for 1 hour at 4C. Chromatin was recovered by centrifugation and sheared to an average size of 400 bp by sonication, giving on average 6 ml of chromatin in FA/SDS. For immunoprecipitations (IPs), 1 µl of affinity purified rabbit anti-Foxo antibody [36] was bound to Protein-G Dynabeads (Invitrogen) and incubated with 450 µl of chromatin for 2 hours at room temperature. Beads were washed three times with FA/SDS, once with TE, and once with 10 mM Tris-HCl pH 8, 250 mM LiCl, 1 mM EDTA, 1% NP40, 0.5% Na Deoxycholate. DNA was recovered, treated with proteases, de-cross-linked, treated with RNase and purified using the Qiagen PCR purification kit (Qiagen). For quantitative PCR, a suitable dilution of total chromatin and IP was used for quantification using the PRISM 7000 sequence-detection system and Power SYBR® Green PCR Master Mix (ABI). For ChIP analysis, relative amounts of the target DNA recovered after ChIP compared to total chromatin were determined using three independent biological replicates. The relative proportion of DNA binding was calculated by dividing the proportion of DNA binding in the ChIP for a single region by the average recovered for all regions for that chromatin to normalise for plate-plate differences.

### Other statistical analyses

Statistical analyses were performed using JMP software (version 4.0.5; SAS Institute). Log rank tests were performed on lifespan and stress survival curves. Other data were tested for normality using the Shapiro-Wilk W test on studentised residuals and where appropriate log-transformed. One-way analyses of variance (ANOVA) and planned comparisons of means were made using Tukey-Kramer HSD test.

## Supporting Information

Figure S1 Stress resistance of *Lnk* mutant flies. (A) Survival of male flies fed 5% hydrogen peroxide. Median survival times are 2.6 days for *w^1118^* (black line; n = 98), 3.7 days for *Lnk^d07478^* (dark blue line; n = 99) and 4.1 days for *Lnk^Del29^* (light blue line; n = 87). (B) Survival of female flies fed 5% hydrogen peroxide. Median survival times are 3.5 days for *w^1118^* (black line; n = 98), 4.6 days for *Lnk^d07478^* (dark red line; n = 99) and 5.6 days for *Lnk^Del29^* (red line; n = 87). (C) Survival of male flies under starvation conditions. Median survival times are as follows: 2.3 days for *w^1118^* (black line; n = 97), 3.6 days for *Lnk^d07478^* (dark blue line; n = 98) and 3.9 days for *Lnk^Del29^* (light blue line; n = 100). (D) Survival of female flies under starvation conditions. Median survival times are 3.6 days for *w^1118^* (black line; n = 93), 5.5 days for *Lnk^d07478^* (dark red line; n = 92) and 5.5 days for *Lnk^Del29^* (red line; n = 88).
(0.63 MB TIF)

Figure S2 Feeding behaviour of *Lnk* mutant flies. Feeding observations of 7 day old flies housed on standard food at a density of 5 flies per vial. Flies were left undisturbed for at least 15 hours before observations were started. Data are presented as the proportion of feeding events/possible feeding events ± SEM. No significant differences were observed in the feeding behavior of *Lnk* mutant flies compared to controls (Males: p = 0.132. Females: p = 0.61. Chi-square test).
(0.19 MB TIF)

Table S1 Functional Categories that are significantly down- or upregulated in *Lnk* mutant microarrays. Catmap analysis was used to identify functional categories associated with genes that show altered expression within *Lnk* mutants compared to controls. For brevity, the full hierarchy of the significant Gene Ontology (GO) categories has not been shown.
(1.11 MB TIF)

Table S2 List of differentially expressed genes (p<0.05; >0.1-fold) in *Lnk* mutants relative to controls. RNA was extracted from the heads of 7-day old females homozygous for the *Lnk^Del29^* allele, along with RNA from age-matched controls. RNA was labelled and hybridised to Affymetrix *Drosophila* 2.0 microarrays. All experiments were conducted in quadruplicate to facilitate statistical analysis. The raw data files were background corrected and normalised by loess normalization and a linear model was fitted to identify a set of differentially expressed genes using the R limma package. The columns show the Affymetrix probe set (A), fold-change in expression level (B), *p*-value (C), FBgn identifier (D) and gene name (E). Genes are sorted by *p*-value.
(0.37 MB XLS)

Table S3 Metabolic pathways affected in *Lnk* mutants. Genes listed in Table S2 were compared to a list of 484 genes that function in *Drosophila* metabolic pathways [35]. The overlap is shown sorted by the metabolic process affected. Red indicates upregulated genes and blue indicates downregulated genes.
(0.04 MB XLS)

Table S4 Genes in the IIS and Ras/MapK pathways that show differential expression in *Lnk* mutants. Genes listed in Table S2 were compared to lists of genes that function in the IIS and Ras/MapK signal transduction pathways. These lists were compiled from gene ontology data as listed in Flybase. dFoxo target genes were previously described (Teleman *et al*. (2008). Nutritional control of protein biosynthetic capacity by insulin via Myc in *Drosophila*. Cell Metabolism 7: 21-32). Genes listed in grey were not reliably detected on the arrays.
(0.05 MB XLS)
