# Supplementary figures and images for: Regulation of Lifespan, Metabolism, and Stress Responses by the Drosophila SH2B Protein, Lnk
Source: PLoS Genet. 2010 Mar 19;6(3):e1000881. doi: 10.1371/journal.pgen.1000881 (PMC2841611; doi:10.1371/journal.pgen.1000881)

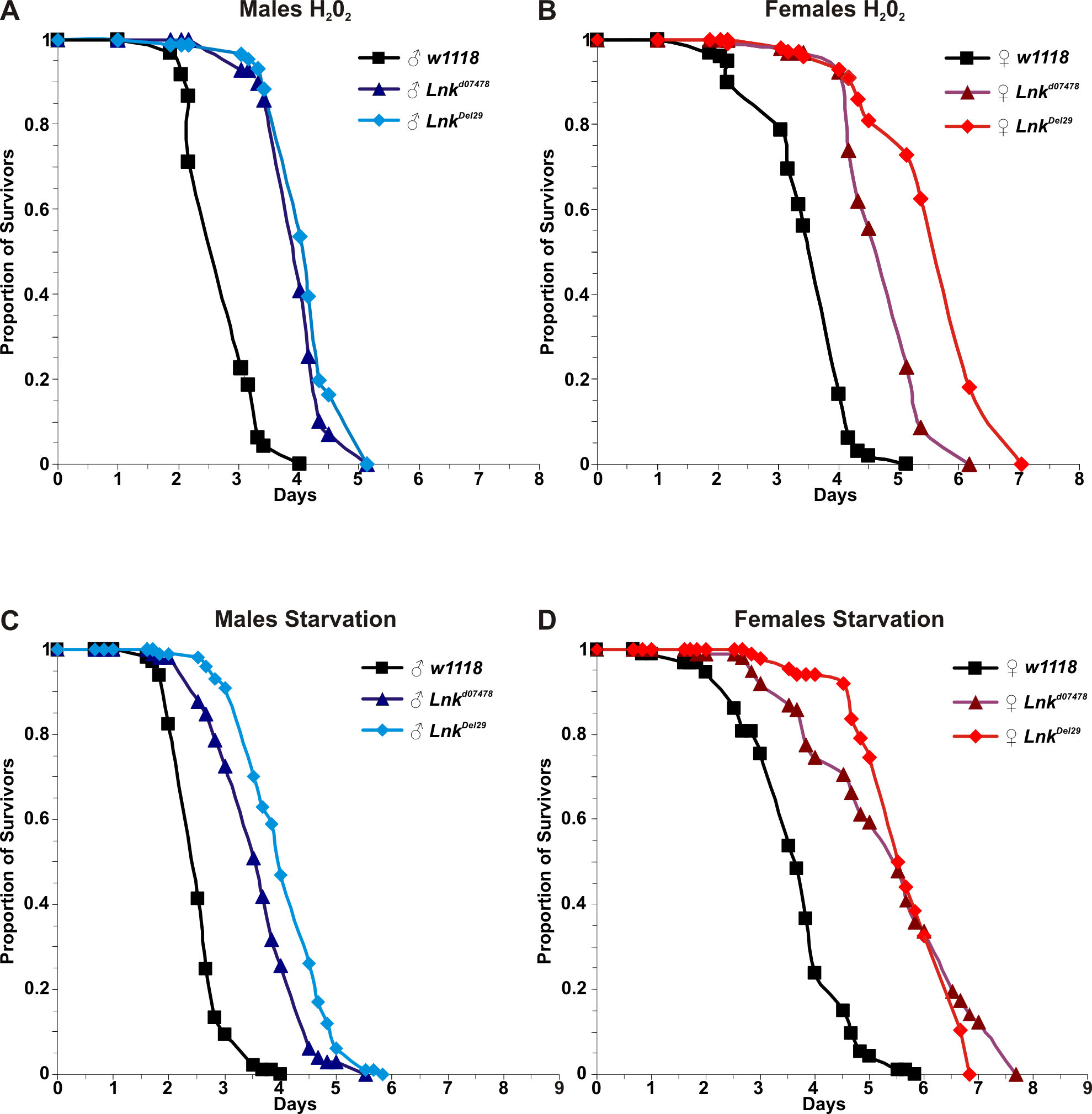

Supplement: Figure S1 — Stress resistance of Lnk mutant flies. (A) Survival of male flies fed 5% hydrogen peroxide. Median survival times are 2.6 days for w1118 (black line; n = 98), 3.7 days for Lnkd07478 (dark blue line; n = 99) and 4.1 days for LnkDel29 (light blue line; n = 87). (B) Survival of female flies fed 5% hydrogen peroxide. Median survival times are 3.5 days for w1118 (black line; n = 98), 4.6 days for Lnkd07478 (dark red line; n = 99) and 5.6 days for LnkDel29 (red line; n = 87). (C) Survival of male flies under starvation conditions. Median survival times are as follows: 2.3 days for w1118 (black line; n = 97), 3.6 days for Lnkd07478 (dark blue line; n = 98) and 3.9 days for LnkDel29 (light blue line; n = 100). (D) Survival of female flies under starvation conditions. Median survival times are 3.6 days for w1118 (black line; n = 93), 5.5 days for Lnkd07478 (dark red line; n = 92) and 5.5 days for LnkDel29 (red line; n = 88). (0.63 MB TIF) [file pgen.1000881.s001.tif]

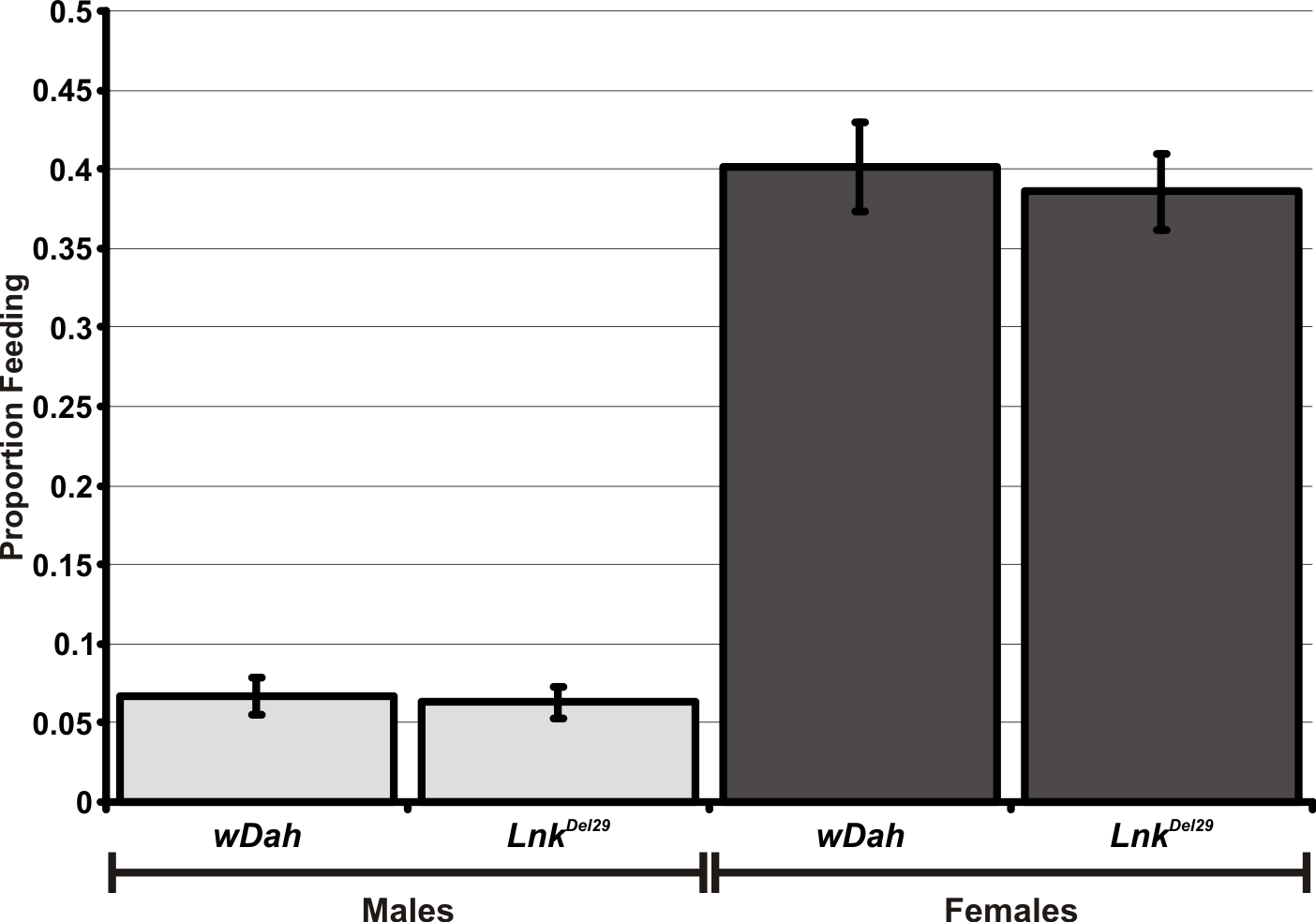

Supplement: Figure S2 — Feeding behaviour of Lnk mutant flies. Feeding observations of 7 day old flies housed on standard food at a density of 5 flies per vial. Flies were left undisturbed for at least 15 hours before observations were started. Data are presented as the proportion of feeding events/possible feeding events ± SEM. No significant differences were observed in the feeding behavior of Lnk mutant flies compared to controls (Males: p = 0.132. Females: p = 0.61. Chi-square test). (0.19 MB TIF) [file pgen.1000881.s002.tif]

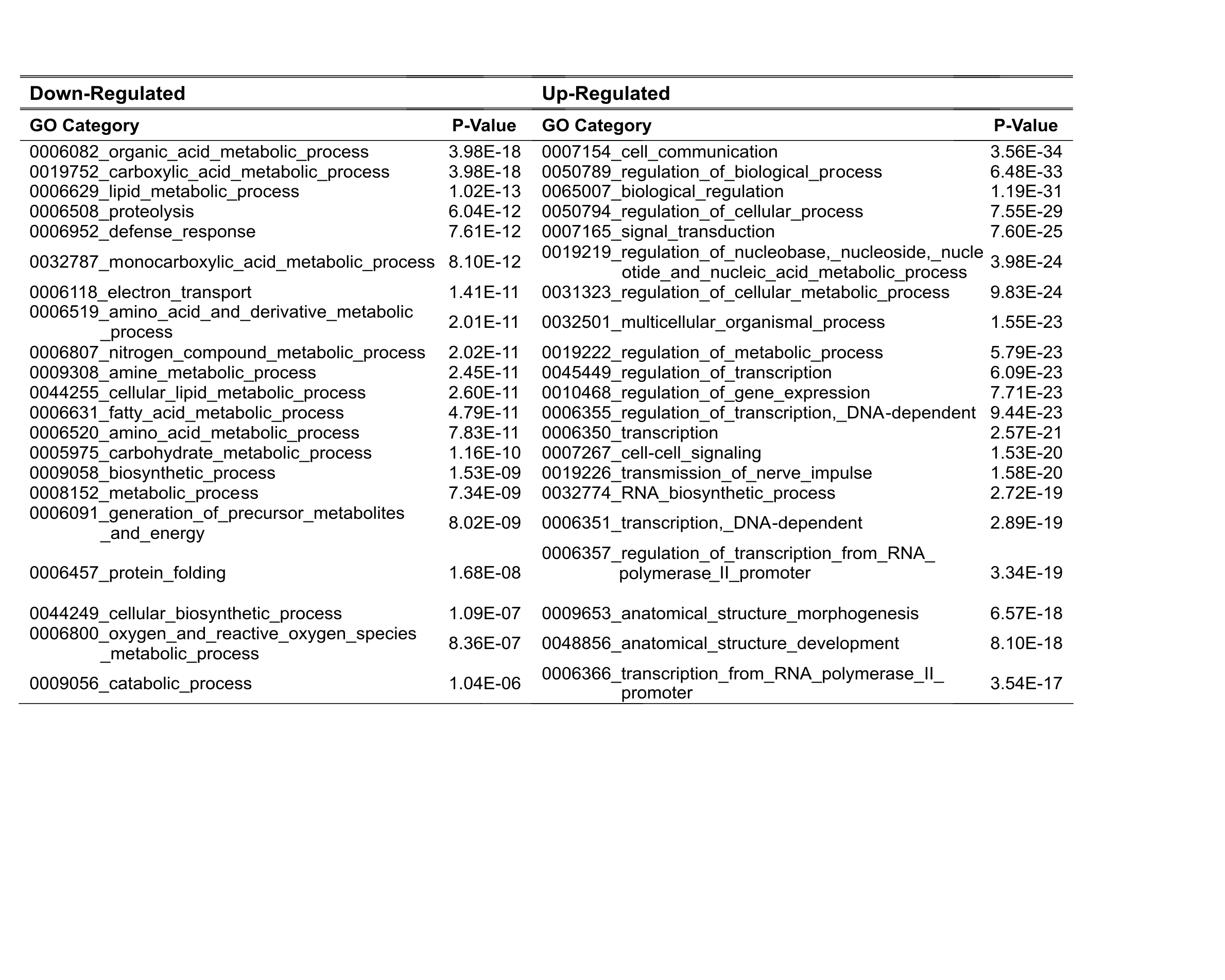

Supplement: Table S1 — Functional Categories that are significantly down- or upregulated in Lnk mutant microarrays. Catmap analysis was used to identify functional categories associated with genes that show altered expression within Lnk mutants compared to controls. For brevity, the full hierarchy of the significant Gene Ontology (GO) categories has not been shown. (1.11 MB TIF) [file pgen.1000881.s003.tif]
